# Natural course of fatty liver in 36,195 South Korean adults

**DOI:** 10.1038/s41598-019-44738-7

**Published:** 2019-07-15

**Authors:** Ki-Chul Sung, Mi-Yeon Lee, Jong-Young Lee, Sung-Ho Lee, Yong-Bum Kim, Won-Jun Song, Ji-Hye Huh, Jin-Sun Park, Jeong-Hun Shin, Mi Hae Seo, Seong-Hwan Kim, Sun H. Kim

**Affiliations:** 1 Division of Cardiology, Department of Internal Medicine, Kangbuk Samsung Hospital, Sungkyunkwan University School of Medicine, Seoul, Republic of Korea; 2 Division of Biostatistics, Department of R&D Management, Kangbuk Samsung Hospital, Sungkyunkwan University School of Medicine, Seoul, Republic of Korea; 3 Department of Neurology, Kangbuk Samsung Hospital, Sungkyunkwan University, School of Medicine, Seoul, South Korea; 4 Department of Critical Care Medicine, Kangbuk Samsung Hospital, Sungkyunkwan University School of Medicine, Seoul, Republic of Korea; 5 Department of Internal Medicine, Yonsei University Wonju College of Medicine, Wonju, Republic of Korea; 6 Department of Cardiology, Ajou University School of Medicine, Suwon, Republic of Korea; 7 Division of Cardiology, Department of Internal Medicine, Hanyang University Guri Hospital, Hanyang University College of Medicine, Guri, Republic of Korea; 8 Division of Endocrinology, Department of Internal Medicine, Gumi Hospital, Soon Chun Hyang University School of Medicine, Gumi, Republic of Korea; 9 Division of Endocrinology, Department of Medicine, Stanford University School of Medicine, Stanford, CA USA

**Keywords:** Cardiology, Risk factors

## Abstract

Nonalcoholic fatty liver disease (NAFLD) is the most common cause of liver disease, and yet the natural course remains unclear. Study population included 36,195 individuals who participated in a health-screening program and diagnosed with fatty liver by abdominal ultrasound. Participants were provided written information regarding fatty liver and advised to make lifestyle changes. Ultrasound was repeated after at least 6 months. After a mean follow up of 4.9 years (±3.4), 19.6% resolved their fatty liver. Individuals who resolved were more likely female (22.9% vs. 12.3%), thinner (body mass index [BMI], 25.2 ± 2.7 vs. 26 ± 2.7), and with lower HOMA-IR (1.4 vs. 1.7) (*P* .70.001). Decrease in BMI predicted resolution of fatty liver with 42% of those in the top quartile of BMI decline resolving compared with 5.7% in the lowest quartile (odds ratio [OR] (95% confidence interval [CI]) 15.65 (14.13–17.34), *P* < 0.001)). Baseline HOMA-IR also predicted resolution with those in the top quartile (most insulin resistant) being least likely to resolve (12%) vs. those in the lowest quartile (25%) (OR 0.36 [0.31–0.42], *P* < 0.001). Fatty liver disease is persistent. Individuals with higher degree of insulin resistance are also the most likely to have persistent steatosis at follow up.

## Introduction

Nonalcoholic fatty liver disease (NAFLD) is the most common cause of liver disease worldwide^1^. Based on a recent meta-analysis, one out of 4 adults is estimated to have NAFLD worldwide^2^. While common, the natural course of NAFLD remains unclear.

Prior studies have focused on highly selected individuals with NAFLD based on retrospective chart review^3–6^. These studies included individuals based on clinical diagnosis of fatty liver^3^, elevated liver enzymes^4^ or liver biopsies^5,6^. As most individuals with fatty liver show no clinical symptoms, signs or laboratory abnormalities, these prior study populations may represent a biased sample with NAFLD.

In South Korea, comprehensive health examinations, including liver ultrasounds, are performed routinely in the general population, and present a unique opportunity to understand the prevalence and natural course of NAFLD in the general population. Individual who receive a diagnosis of fatty liver are given information about the association of obesity with fatty liver and advised to moderate calories (by limiting carbohydrate, sugar, fried foods, and alcohol) and to exercise regularly.

The purpose of this study is to present follow-up on 36,195 individuals with diagnosis of fatty liver by ultrasound. This is the largest known cohort of individuals with fatty liver^2^. The specific aims were to evaluate resolution of fatty liver at follow up and predictors of resolution. Specifically, we evaluated the effects of obesity and insulin resistance, both strong predictors of fatty liver^7,8^, on resolution of steatosis.

## Results

As can be seen in Table 1, the overall group was young with a mean age of 37, and more than half were obese (BMI ≥ 25 kg/m^2^) at baseline. Fatty liver persisted in a majority of individuals at follow and only 19.6% resolved their fatty liver. Those with resolution were slightly older. A greater proportion of women resolved than men (31% vs. 18%). Those with resolution were also more likely to be thinner with better metabolic profile (lower glucose, insulin, HOMA-IR, low-density lipoprotein [LDL], and triglyceride, and higher high-density lipoprotein cholesterol [HDL-C]) and lower prevalence of prediabetes and hypertension at baseline. They were also slightly less likely to have education beyond high school and less likely to be current smokers. NAFLD fibrosis score was overall low but slightly higher in those who resolved their fatty liver at follow up. This difference is likely due to higher age and lower albumin in those who resolved. Incidence for resolution of fatty liver /100 person-years of follow-up was 3.97 (Male: 3.52, Female: 6.94) (Table 2).

**Table 1:** Baseline characteristics based on resolution of fatty liver.

	Resolved fatty liver	Persistent fatty liver	*P* value
N	7,086	29,109	<0.001
Age (years)	37.1 ± 7.1	36.7 ± 6.9	<0.001
Female, *n* (%)	1,616 (22.9)	3,588 (12.3)	<0.001
BMI (kg/m^2^)	25.2 ± 2.7	26.0 ± 2.7	<0.001
Education, *n* (%)			<0.001
≤high school	822 (11.6)	2,853 (9.8)	
>high school	3,993 (56.3)	17,143 (58.9)	
Unknown	2,271 (32.1)	9,113 (31.3)	
Exercise, *n* (%)			0.663
<1 time per week	3,932 (55.5)	16,069 (55.2)	
≥1 time per week	3,154 (44.5)	13,040 (44.8)	
Smoking, *n* (%)			<0.001
Never/former	4,849 (68.4)	17,952 (61.7)	
Current	2,138 (30.2)	10,670 (36.7)	
Unknown	99 (1.4)	487 (1.7)	
Glucose (mg/dL)	95.8 ± 13.4	96.1 ± 12.6	0.043
Insulin (µIU/mL)^a^	5.9 (4.2–8.2)	7.1 (5.1–9.8)	<0.001
HOMA-IR^a^	1.4 (1.0–2.0)	1.7 (1.2–2.4)	<0.001
LDL-C(mg/dL)	124 ± 29	129 ± 30	<0.001
Triglyceride (mg/dL)	130 (94–182)	149 (109–207)	<0.001
HDL(mg/dL)	51 ± 11	48 ± 10	<0.001
AST (IU/L)	23 (19–28)	25 (21–32)	<0.001
ALT (IU/L)	27 (19–37)	34 (24–49)	<0.001
Albumin (g/dL)	4.6 ± 0.2	4.7 ± 0.2	<0.001
Platelet (10^3^/mm^3^)	261 ± 56	261 ± 53	0.7634
hs-CRP(mg/dl)	0.06 (0.03–0.12)	0.07 (0.04–0.14)	<0.001
Obese, *n* (%)^b^	3,579 (50.5)	18,029 (61.9)	<0.001
Prediabetes, *n* (%)	1,769 (25.0)	8,134 (27.9)	<0.001
Diabetes, *n* (%)	181 (3.9)	655 (3.3)	0.051
Hypertension, *n* (%)	87 0 (12.3)	4.009 (13.8)	0.001
CVD, *n* (%)	210 (3.0)	758 (2.6)	0.092
NAFLD Fibrosis Score^c^	−3.15 ± 1.0	−3.22 ± 1.0	<0.001

Data are mean ± standard deviation or median (interquartile range) unless otherwise specified.

^a^Insulin concentration and HOMA-IR were available for 13, 688 individuals.

^b^Obesity was defined by a BMI ≥25 kg/m^2^.

^c^NAFLD fibrosis score = −1.675 + 0.037 × age (years) + 0.094 × BMI (kg/m^2^) + 1.13 × IFG/diabetes (yes = 1, no = 0) + 0.99 × AST/ALT ratio − 0.013 × platelet (×10^9^/l) − 0.66 × albumin (g/dl).

*AST* aspartate transaminase, *ALT* alanine aminotransferase, *BMI* body mass index, *CI* confidence interval, *CVD* cardiovascular disease, *HDL* high-density lipoprotein, *HOMA-IR* homeostatic model assessment-Insulin resistance, *hs-CRP* high-sensitivity c-reactive protein, *LDL* low-density lipoprotein, *NAFLD* nonalcoholic fatty liver disease, *OR* odds ratio.

**Table 2:** Incidence for resolution of fatty liver/100 person-years of follow-up.

	n	Person-years	Incidence case	Incidence rate (100 person-years)
All	36,195	178484.5	7,086	3.97
Male	30,991	155201.1	5,470	3.52
Female	5,204	23283.36	1,616	6.94

**Figure 1:**
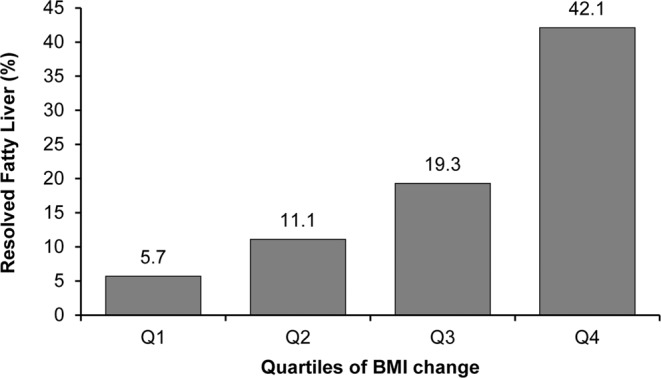
Resolution of fatty liver by BMI change. Q4 had the greatest decline in BMI at follow up.

We evaluated difference in BMI change between those with and without resolution of fatty liver. Those who resolved had a decrease in BMI compared with an increase in those with persistent fatty liver (mean ± standard deviation [SD], −1.0 ± 1.5 vs. 0.2 ± 1.2, *P* < 0.001). We further evaluated the resolution of fatty liver by quartiles of BMI change. As seen in Fig. 1, 42% of those who had the greatest decline in BMI (Q4) resolved their fatty liver at follow up. BMI change remained a significant predictor of fatty liver resolution in a multiple regression analysis, adjusted for age, sex, baseline BMI, education, exercise, smoking and alcohol intake (Table 3). BMI change remained significant when stratified by sex.

**Table 3:** OR (95% CI) for fatty liver resolution by BMI change^a^ quartiles.

BMI Change	Q1 (8.36 to 0.73) (*n* = 9,038)	Q2 (0.72 to 0.01) (*n* = 9,041)	Q3 (−0 to 0.75) (*n* = 9,036)	Q4 (−0.76 to −13.03) (*n* = 9,080)	*P* for trend
Resolved fatty liver, *n* (%)	517 (5.7)	1,005 (11.1)	1,739 (19.3)	3,825 (42.1)	<0.001
All	1 (reference)	2.13 (1.91–2.39)	4.30 (3.87–4.78)	15.65 (14.13–17.34)	<0.001
Male	1 (reference)	2.12 (1.86–2.42)	4.48 (3.96–5.07)	16.18 (14.36–18.23)	<0.001
Female	1 (reference)	2.34 (1.87–2.94)	3.82 (3.07–4.75)	14.49 (11.74–17.87)	<0.001

Adjusted for age, sex, baseline BMI, education, exercise, smoking and alcohol intake (g/day).

^a^BMI change was calculated as BMI_follow-up_ − BMI_baseline_.

*BMI* body mass index, *CI* confidence interval, *OR* odds ratio.

Insulin concentration and HOMA-IR were available for 13,688 individuals (38%). In this subgroup, BMI change remained significantly associated with fatty liver resolution when adjusted for HOMA-IR (Supplementary Table 1). We also evaluated the association between baseline HOMA-IR and resolution of fatty liver (Table 4) to evaluate the effect of insulin resistance to predict fatty liver change. The most insulin-resistant quartile (Q4) were the least likely to resolve their fatty liver. Only 12% resolved compared with 25% in the most insulin-sensitive quartile (Q1). Thus, the OR for fatty liver resolution declined as HOMA-IR increased and this was similar for both men and women, after adjusting for covariates. Table 4 also shows the baseline BMI and BMI change by quartile of HOMA-IR. Baseline BMI increased from HOMA IR Q1 to Q4. Interesting, BMI change declined from Q1 to Q4. Thus, average BMI was more likely to decrease in the HOMA-IR Q4 than Q1, although resolution of fatty liver was lowest in Q4.

**Table 4:** OR (95% CI) for fatty liver resolution by HOMA-IR quartiles.

HOMA-IR	Q1 (0.07 to 1.13) (*n* = 3,422)	Q2 (1.13 to 1.62) (*n* = 3,420)	Q3 (1.63 to 2.31) (*n* = 3,424)	Q4 (2.31 to 18.13) (*n* = 3,422)	*P* for trend
BMI baseline	24.5 ± 2.3	25.4 ± 2.5	26.1 ± 2.6	27.6 ± 3.2	<0.001
BMI change^a^	0.17 ± 1.14	0.07 ± 1.21	0.04 ± 1.28	−0.09 ± 1.47	<0.001
Resolved fatty liver, *n* (%)	850 (24.8)	699 (20.4)	508 (14.8)	409 (12.0)	<0.001
All	1 (reference)	0.77 (0.68 to 0.88)	0.50 (0.43 to 0.57)	0.36 (0.31 to 0.42)	<0.001
Male (*n* = 11,724)	1 (reference)	0.80 (0.70 to 0.92)	0.50 (0.43 to 0.59)	0.35 (0.29 to 0.42)	<0.001
Female (*n* = 1,964)	1 (reference)	0.56 (0.40 to 0.77)	0.42 (0.30 to 0.58)	0.33 (0.24 to 0.46)	<0.001

Adjusted for age, sex, baseline BMI, BMI change, education, exercise, smoking and alcohol intake (g/day).

^a^BMI change was calculated as BMI_follow-up_−BMI_baseline_.

*BMI* body mass index, *CI* confidence interval, *HOMA-IR* homeostatic model assessment-Insulin resistance, *OR* odds ratio.

## Discussion

The global prevalence of NAFLD is 25%^2^, which is similar to the estimate in our population of South Korean adults. After a mean follow-up of 4.9 years, approximately 1 in 5 adults resolved their fatty liver. The individuals who resolved were more likely female, less insulin resistant, and with fewer metabolic abnormalities. In addition, those who resolved were more likely to have lost weight.

NAFLD reflects a spectrum of disease from steatosis alone to steatohepatitis. Our study population had low NAFLD Fibrosis Score, and most likely had steatosis alone. Although individuals with steatohepatitis are at highest risk to progress to fibrosis and cirrhosis^1^, individuals with steatosis alone can also develop progressive hepatic fibrosis^9^. Insulin resistance is important to the pathogenesis and progression of NAFLD^10,11^. Our study population was selected to have fatty liver disease by ultrasound and likely more insulin resistant than individuals without fatty liver disease^7^. Even in this population selected for fatty liver, the degree of insulin resistance was negatively associated with fatty liver resolution at follow up. Thus, those in the highest HOMA-IR quartile were 64% less likely to resolve their fatty liver compared with the lowest HOMA-IR quartile.

As expected, higher HOMA-IR was associated with higher BMI. Interestingly, individuals with higher HOMA-IR had greater decline in BMI, albeit modest, at follow-up. This paradox likely reflects the ineffectiveness of modest weight loss and not to inherent futility of weight loss in insulin-resistant individuals. Wong *et al*. showed in a Hong Kong population with fatty liver that less than 3% weight loss was associated with 13% resolution of NAFLD versus 97% resolution in those who lost at least 10% of baseline weight^12^. Their population had a similar mean BMI ~25 kg/m^2^ as ours. Furthermore, prior studies have supported that individuals with fatty liver with more unfavorable metabolic risk factors at baseline (e.g., hyperglycemia) require greater degree of weight loss to resolve fatty liver^13^. The mean decline in BMI in the HOMA-IR Q4 was 0.09 kg/m^2^. Thus, a male weighing 79.5 kg pounds with a height of 1.7 meters would have lost only 0.3% of his baseline weight. Thus, mean weight loss was minor.

There are limitations to our study. We diagnosed fatty liver based on ultrasound. Ultrasound has been shown to be reliable and accurate for detecting moderate to severe fatty liver^14^. However, ultrasound may miss mild degree of fatty liver. Nevertheless, ultrasound provides a noninvasive and feasible means to evaluate fatty liver in a large population.

In conclusion, we show in a large cohort of individuals that fatty liver disease is persistent. Unfortunately, individuals with the greatest risk for progressive liver disease—those with higher degree of insulin resistance and metabolic abnormalities—are also the most likely to have persistent steatosis at follow up. Weight loss, as confirmed in this study, remains the major intervention to resolve fatty liver. However, usual care is likely not sufficient to motivate major weight loss.

## Materials and Methods

The study population consisted of individuals who participated in a comprehensive health-screening program, at least twice, at Kangbuk Samsung Hospital, Seoul and Suwon, Korea from 2002 to 2014 (*n* = 259,011). Fatty liver, as determine by ultrasound, was present at baseline in 67,138 individuals (25%). Individuals with fatty liver were excluded from the study if they were less than 20 years old and consumed >30 g/day (men) or >20 g/day (women) of alcohol. We also excluded individuals who were receiving treatment for diabetes, hypertension, or hyperlipidemia (including statin treatment). Individuals were also excluded for potential secondary etiologies of liver disease: positive hepatitis C antibody status (*n* = 34 at baseline and *n* = 69 at follow up); positive hepatitis B surface antigen status (*n* = 840 at baseline and *n* = 1,753 at follow up); and evidence of cancer (*n* = 682 at baseline and *n* = 1,675 at follow up). Individuals were also excluded for missing data (*n* = 11,299 at baseline and *n* = 12,642 at follow up). After these exclusions, the final number of study participants was 36,195. Mean follow up was 4.93 years (±3.39); median was 3.94 years (maximum 12.65 years). The Institutional Review Board of Kangbuk Samsung Hospital approved the study and waived informed consent as de-identified information was retrieved retrospectively.

### Measurements

As part of the health-screening program, individuals completed self-administered questionnaires, related to their medical and social history and medication use. Individuals were asked about duration of education (years), smoking history (never, former, or current) and alcohol consumption (g/day). We also assessed frequency of moderate- or vigorous-intensity physical activity per week.

Trained staff collected anthropometric measurements and vital statistics. Body weight was measured in light clothing with no shoes to the nearest 0.1 kilogram using a digital scale. Height was measured to the nearest 0.1 centimeter. Body mass index (BMI) was calculated as weight in kilograms divided by height in meters squared. Blood samples were collected after at least 10-hours of fasting and analyzed in the same core clinical laboratory. The core clinical laboratory has been accredited and participates annually in inspections and surveys by the Korean Association of Quality Assurance for Clinical Laboratories.

Insulin concentrations were available for 13,688 individuals (38%). Homeostatic model assessment of insulin resistance (HOMA-IR) was used as a surrogate measure of insulin resistance and calculated using the following equation: $\frac{{fasting}\,{insulin}(\frac{{mU}}{{L}}){xfasting}\,{glucose}(\frac{mmol}{{L}})}{22.5}$^15^. The study population was divided into quartiles of HOMA-IR.

Clinical radiologists performed abdominal ultrasonography (Logic Q700 MR; GE, Milwaukee, WI, USA) using a 3.5 MHz probe for all participants at baseline and follow-up. The following images were undertaken: i) sagittal view of the right lobe of the liver and right kidney, ii) transverse view of the left lateral segment of the liver and spleen and iii) transverse view of the liver for altered echo texture. Fatty infiltration of the liver (fatty liver) was identified if there was an increase in echogenicity of the liver compared with the echogenicity of the renal cortex where the diaphragm and intrahepatic vessels appeared normal. Individuals diagnosed with fatty liver were provided a handout about the association between fatty liver and obesity. They also were advised to maintain normal weight, reduce caloric intake, moderated carbohydrates, sugar, fried foods and alcohol, and get regular exercise to reduce fat in the liver.

### Statistical analyses

The statistical analysis was performed using STATA version 15.0 (StataCorp LP, College Station, TX, USA). Reported *P* values were two-tailed, and <0.05 were considered statistically significant. The distribution of continuous variables was evaluated, and transformations were conducted for nonparametric variables. We conducted multiple linear regression analyses to evaluate the effect of change in BMI (from baseline to follow-up) on resolution of fatty liver at follow-up. In the subset with insulin concentration, we also evaluated baseline insulin resistance (HOMA-IR) to predict fatty liver resolution. Analyses were adjusted for age, BMI, sex, education, exercise, smoking and alcohol intake.

## Supplementary information

OR (95% CI) for fatty liver resolution by BMI change quartile in those with HOMA-IR
