# Supplementary material for: Natural course of fatty liver in 36,195 South Korean adults
Source: Sci Rep. 2019 Jul 15;9:9062. doi: 10.1038/s41598-019-44738-7 (PMC6629682; doi:10.1038/s41598-019-44738-7)
Supplement: Supplementary file 1 — OR (95% CI) for fatty liver resolution by BMI change quartile in those with HOMA-IR [file 41598_2019_44738_MOESM1_ESM.pdf]

Natural course of fatty liver in 36,195 South Korean adults

Ki-Chul Sung, Mi-Yeon Lee, Jong-Young Lee, Sung-Ho Lee, Yong-Bum Kim, Won-Jun Song, Ji- Hye Huh, Jin-Sun Park, Jeong-Hun Shin, Mi Hae Seo, Seong-Hwan Kim & Sun H. Kim

Supplementary Table 1. OR (95% CI) for fatty liver resolution by BMI change quartile in those with HOMA-IR

**BMI Change**	**Q1 (8.36 to 0.73) (n = 3,519)**	**Q2 (0.72 to 0.01) (n = 3,829)**	**Q3 (0 to -0.75) (n = 3,477)**	**Q4 (-0.76 to -13.03) (n = 2,863)**	***p* for trend**
Resolved fatty liver, n (%)	205 (8.3)	430 (17.4)	656 (26.6)	1,175 (47.7)	<0.001
All	1 (reference)	2.13 (1.78 - 2.54)	4.38 (3.69 - 5.19)	16.32 (13.77 - 19.34)	<0.001
Male (n = 11,724)	1 (reference)	2.13 (1.72 - 2.62)	4.45 (3.64 - 5.43)	16.91 (13.88 - 20.61)	<0.001
Female (n = 1,964)	1 (reference)	2.27 (1.60 - 3.23)	4.26 (3.02 - 6.02)	14.54 (10.31 - 20.51)	<0.001

Adjusted for age, sex, baseline BMI, HOMA-IR, education, exercise, smoking and alcohol intake (g/day).

BMI, body mass index; CI, Confidence interval; HOMA-IR, homeostatic model assessment-Insulin resistance; OR, odds ratio.
